# Simvastatin Improves the High-Fat-Diet-Induced Metabolic Disorder in Juvenile Chinese Giant Salamander (*Andrias davidianus*) Through Inhibiting Endoplasmic Reticulum Stress and Enhancing Mitochondrial Function

**DOI:** 10.3390/ani16010134

**Published:** 2026-01-02

**Authors:** Yuheng Wang, Jun Chen, Yanzou Dong, Jie Du, Sisi Ma, Huicong Wang, Yaoyue Wang, Xiangfei Li

**Affiliations:** 1 Department of Animal Husbandry and Veterinary Medicine, Jiangsu Vocational College of Agriculture and Forestry, Jurong 212400, China; yuhengyg@163.com (Y.W.);; 2 Key Laboratory of Aquatic Nutrition and Feed Science of Jiangsu Province, College of Animal Science and Technology, Nanjing Agricultural University, No. 1 Weigang Road, Nanjing 210095, China

**Keywords:** high-fat diet, endoplasmic reticulum stress, mitochondrial function, simvastatin, *Andrias davidianus*

## Abstract

In order to deeply understand the potential mechanisms underlying the metabolic disorders of Chinese giant salamander (*Andrias davidianus*), a total of two feeding trials were conducted in the present study. For experiment I, the diets containing five graded levels of lipids at 32.8, 58.7, 87.9, 122.4, and 149.2 g/kg were formulated, respectively, and fed to juvenile *A. davidianus* for 90 days. The quadratic regression analysis based on growth performance results indicated that the optimal dietary lipid level is 95.16–101.02 g/kg. Meanwhile, a dietary lipid level of 149.2 g/kg was found to reduce the growth performance of *A. davidianus*. Based on this, in experiment II, a normal-fat diet (86.8 g/kg crude lipid), a high-fat diet (HFD, 148.4 g/kg crude lipid), and an HFD supplemented with 0.1 g/kg simvastatin were prepared, respectively, and fed to the juveniles for 90 days. The results indicated that HFD feeding resulted in hyperlipidemia, hepatic damage, endoplasmic reticulum stress, and mitochondrial dysfunction, while simvastatin administration alleviated these symptoms. In conclusion, simvastatin could alleviate the HFD-induced metabolic disorders in *A. davidianus*, as may be achieved by inhibiting ER stress and enhancing mitochondrial function.

## Simple Summary

Chinese giant salamander (*Andrias davidianus*), an emerging aquatic economic species, frequently exhibits metabolic disorders in practical culture due to the adoption of formulated feed. To date the underlying mechanisms are still poorly elucidated with the nutritional interventions poorly developed. As feeding a high-fat diet is a well-established approach for modeling metabolic disorders, we first evaluated the optimal dietary lipid requirement (95.16–101.02 g/kg) of *A. davidianus* based on growth performance. Subsequently, a high-fat-diet (149.2 g/kg lipid)-induced metabolic disorder model was established. Meanwhile, given its established lipid-lowering properties, simvastatin was used as a nutritional intervention to alleviate the metabolic disorders. As a result, simvastatin supplementation effectively alleviated the liver damage and hyperlipidemia of *A. davidianus* fed a high-fat diet by inhibiting endoplasmic reticulum stress and enhancing mitochondrial function. Our findings partly unveil the pathological basis of the metabolic disorders in *A. davidianus* and provide new insights for the prevention and cure of it.

## 1. Introduction

Amphibians have a special classification status and present a particular significance in terms of biology, ecology, and evolution studies [1]. Chinese giant salamander (*Andrias davidianus*), colloquially called “baby fish”, is the world’s largest extant amphibian with an evolutionary history spanning over 350 million years [2]. Despite its listing on the IUCN Red List, *A. davidianus* has emerged as an economically significant species due to the successful artificial breeding programs [3]. *A. davidianus* possesses significant nutritional values, as it is rich in protein, essential amino acid, mineral elements, and docosahexaenoic acid. Its muscle and skin are also found to have abundant bioactive components, including collagen, bioactive peptides, bombesin, etc., all of which display anti-fatigue, anti-aging, anti-tumor, and anti-infection effects. Therefore, *A. davidianus* enjoys the reputation of being “aquatic ginseng” [3]. In recent years, the market demand and artificial breeding scale for *A. davidianus* have continually grown in China. To date, the farming area for this species covered 16,208 hectares in China in 2024, representing an 312% increase from 2015 [4].

However, as the production increases, the market price of *A. davidianus* exhibits a decreasing trend [5]. Thus, investigating the approaches to reduce the production cost is of great significance for ensuring the sustainable culture of this species. Application of artificial formula feed could effectively decrease the cost of animal farming [6]. Considering this, artificial compound feed is increasingly adopted in the practical culture of *A. davidianus*. However, this unfortunately results in the frequent occurrence of liver disease marked by metabolic disorders [3,5]. This may be ascribed to the unreasonable nutritional composition in diets, since unsuitable nutritional status would damage the function of liver. As we all know, liver is a central organ for homeostasis, detoxification, and immunity. Thus, liver damage would pose a significant influence on the health status of the body [6]. Accordingly, compromised liver function has been reported to induce a series of adverse consequences in aquatic species including metabolic disturbance, immune dysregulation, and slow growth [7,8]. Nowadays the nutritional and pharmacological interventions for liver diseases have been extensively reported in mammals and other vertebrates. However, relevant information is still quite lacking in Amphibians. Hence, it is urgent to explore the inner mechanisms underlying the metabolic disorders in *A. davidianus* so as to promote targeted nutritional approaches to alleviate the diet-induced liver damage.

The endoplasmic reticulum (ER) and mitochondria engage in extensive physical and functional coupling, forming a critical signaling hub that regulates cellular metabolism and homeostasis [9,10]. ER stress in hepatocytes is a hallmark of the pathophysiology of metabolic disease, as could be activated by the unsuitable nutritional status such as lipid overload [11,12]. This action perturbs lipid metabolism and compromises mitochondrial function. The resulting hepatic metabolic dysregulation and injury contribute significantly to the decline in overall health and feed utilization of animals [13,14,15].

Simvastatin is a classical lipid metabolism regulator. Administered orally as a prodrug in its lactone form, simvastatin undergoes a rapid first-pass hydrolysis in the liver into its active β-hydroxy acid metabolite, thereby regulating metabolic processes [16,17]. It is widely accepted that simvastatin is an effective drug for metabolic diseases, including dyslipidemia, fatty liver, and coronary heart disease in mammals [18]. Its benefits have been found to be related to the inhibition of lipid catabolism and the promotion of lipid transportation [19,20]. Based on these facts, simvastatin might be used to alleviate the diet-induced metabolic disorders in *A. davidianus*. Feeding a high-fat diet (HFD) is a generally accepted method to establish metabolic disorder models. However, the lipid content of the HFD must be carefully determined according to the species’ dietary lipid requirement [21,22,23]. Considering this, the optimal dietary lipid requirement of *A. davidianus* was evaluated by feeding a series of diets containing graded levels of lipid firstly (experiment I) in this study. Based on the obtained results, an HFD was formulated to induce the metabolic dysfunction in this species, with a normal-fat diet (NFD) group serving as the control. In addition, alleviating action of simvastatin was also tested focusing on ER stress and mitochondrial function (experiment II). The findings of the present study provide new insights for the prevention and cure of metabolic disorders in *A. davidianus* fed artificial formula feed, thereby promoting the sustainable aquaculture of it and other similar species.

## 2. Materials and Methods

### 2.1. Diets, Animals, and the Feeding Trial

For experiment I, the diets containing five graded levels of lipids at 32.8, 58.7, 87.9, 122.4, and 149.2 g/kg were formulated (Table 1). For experimental II, an NFD (86.8 g/kg lipid) was papered as the control diet, and a diet contained 148.4 g/kg crude lipid was regarded as HFD. The simvastatin (MK 77333, ≥99% in purity) was purchased from MedChemExpress (Shanghai, China) and added to the HFD at the dose of 100 mg/kg (HFD_S, Table 2). This dose is referred to in a previous study, in which simvastatin could alleviate the metabolism disorders of the HFD-fed fish without obvious side effects [14]. All solid raw ingredients were pulverized and sieved through sieve #40, mixed well, and blended with soybean oil and fish oil. The obtained mixture was then mixed with water to make a dough, which was pelleted into the three-millimeter-diameter pellets. Following drying at 60 °C, the pellets were maintained at −20 °C until required for use.

The juvenile *A. davidianus* were obtained at Jurong Longquan giant salamander breeding center (Zhenjiang, China) and transported to an indoor aquaculture system at Jiangsu Vocational College of Agriculture and Forestry (Jurong, China) for a two-week acclimation period. After that, the healthy *A. davidianus* with similar body weights (44.25 ± 0.30 g for experiment I, 44.32 ± 0.23 g for experiment II) were randomly divided into 27 (15 for experiment I, 9 for experiment II) plastic tanks (0.5 × 0.5 × 0.45 m, L × W × H) with a density of 10 individuals per tank; each experimental diet was randomly allocated to three tanks. Both feeding trials lasted for 90 days. The salamanders were fed to satiation once a day at 15:00 h, and the residual diets were collected, dried, weighted, and excluded from the calculation of food intake. During the acclimation period and feeding experiments, the water was completely replaced every day and maintained at the following indices: temperature = 19.0 ± 2.0 °C, dissolved oxygen = 6.0 ± 1.0 mg/L, pH = 7.5 ± 0.3.

### 2.2. Sampling

The salamanders were first fasted for 24 h before sampling and then captured, counted, and weighed from each tank for the assessment of growth performance. After that, five individuals from each tank were randomly selected and anesthetized using 100 mg/L tricaine methanesulfonate solution. Blood was drawn from the caudal vessels using heparinized 1 mL syringes. After centrifugation at 4 °C (4000× *g*, 10 min), plasma was obtained from the supernatant and transferred to −80 °C for subsequent analysis. Following dissection, liver was isolated, rinsed with ice-cold phosphate-buffered saline (PBS; 0.01 M, pH 7.4), and transferred to −80 °C after being quick-frozen (in liquid nitrogen).

### 2.3. Proximate Composition Analysis

Diet proximate composition (moisture, crude protein, and crude lipid) was tested following the AOAC-documented methods [24]. Specifically, the moisture content was determined by oven-drying the samples at 105 °C to constant. The crude protein was quantified by Kjeldahl’s method, and the crude protein level was calculated based on N × 6.25. The crude lipid was extracted via the Soxhlet extractor method with petroleum ether. Ash level was determined gravimetrically by using a muffle furnace at 550 °C.

### 2.4. Plasma Biochemical Parameters Analysis

For plasma biochemical indices, the activities of aspartate aminotransferase (AST) and alanine aminotransferase (ALT) were assayed by the colorimetric method based on the reactions with α-Ketoglutarate [25]. The content of total protein, triglyceride (TAG), total cholesterol (TC), low density lipoprotein (LDL), and high density lipoprotein (HDL) was all determined by an AU5800 automatic biochemical analyzer (Beckman Coulter, Brea, CA, USA). Moreover, the activities of alkaline phosphatase (A059-2, AKP), superoxide dismutase (A001-3, SOD (http://www.njjcbio.com/products.asp?id=286 (accessed on 15 May 2023))), and catalase (A007-1, CAT) were both assayed using commercial assay kits (Nanjing Jiancheng, Nanjing, China).

Differential centrifugation methods were used to prepare the hepatic mitochondria, microsomes, and cytostome samples. Briefly, liver homogenate was made by homogenizing 0.1 g of liver in 1.0 mL Tris-HCl buffer. Then, the homogenate was centrifuged at 1000 rpm for 10 min, and the resulting supernatant was subsequently centrifuged at 8000 rpm for 15 min. The precipitate was mitochondria sample. The supernatant was collected again for the next centrifugation (6000 rpm, 30 min). The final precipitate is microsomes, and the final supernatant is cytostome sample. After that, the succinate dehydrogenase (A022-1, SDH) and Na^+^, K^+^-ATPase activity (A070-2 (http://www.njjcbio.com/products.asp?id=391 (accessed on 15 May 2023))) in the mitochondria, Ca^2+^-ATPase activity (A070-4) in the microsomes, and cytochrome C content (A090-1) in the cytostome were tested, respectively, by commercial kits manufactured by Nanjing Jiancheng.

### 2.5. Gene Expression

Total RNA was extracted from liver tissues using the TRIzol method. The purity of the RNA sample was verified spectrophotometrically (NanoDrop 2000, Thermo Fisher, Waltham, MA, USA) with its integrity confirmed by electrophoresis. Then, RNA sample was reverse-transcribed using a commercial kit (ZR102, Zomanbio, Beijing, China) under manufacturer-recommended conditions. After that, quantitative real-time PCR (qPCR) analysis was conducted on a QuantStudio 6 Flex system (Applied Biosystems, Waltham, MA, USA) with 2xHQ SYBR qPCR Mix (Zomanbio). The specific primers (designed via Primer 5.0 software; sequences in Table 3) were used at 200 nM final concentration. Amplification program was set as the following parameters: 95 °C for 30 s; 40 cycles of 95 °C for 10 s, 60 °C for 30 s. The 2^−ΔΔCt^ method was used to calculate relative gene expression values, and *β-actin* was selected as the internal control gene.

### 2.6. Statistical Analysis

All data were expressed as mean ± S. E. The normality of the data and the homogeneity of variances were assessed with the Shapiro–Wilk test and Levene’s test, respectively. The differences were analyzed by one-way ANOVA followed by Tukey’s post hoc test in experiment I, and Students’ *t* test in experiment II, respectively, under the SPSS 22.0 program. The significant difference was considered when *p* < 0.05. Furthermore, quadratic regression analysis was adopted to estimate the optimal dietary lipid level of *A. davidianus*, as was performed under GraphPad Prism 9.3 program.

## 3. Results

### 3.1. Experiment I: Evaluation of the Optimal Lipid Requirement

As shown in Table 4, survival exhibited no significant difference among all groups (*p* > 0.05). The final body weight (FBW), weight gain rate (WGR), specific growth rate (SGR), and feed intake (FI) all increased significantly as dietary lipid levels increased from 32.8 to 87.9 g/kg, then decreased significantly with further increasing lipid contents (*p* < 0.05). However, feed efficiency ratio (FCR) showed an opposite result (*p* < 0.05) and showed no statistical difference when dietary lipid levels exceeded 87.9 g/kg (*p* < 0.05). Furthermore, condition factor (CF) presented no marked difference (*p* > 0.05), while the values of hepatosmatic index (HSI) increased with the increasing dietary lipid level (*p* < 0.05).

**Figure 1:**
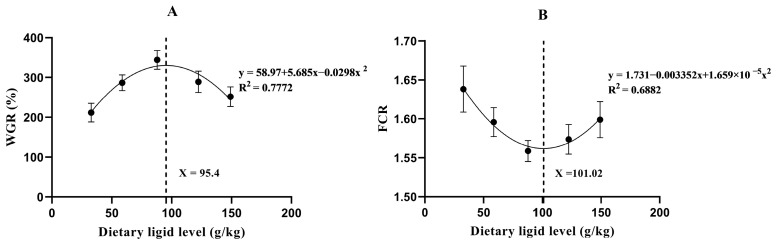
The optimal dietary lipid levels for *A. davidianus* derived from the secondary regression models of WGR (**A**) and FCR (**B**). The dashed lines illustrate optimal dietary lipid level.

The further quadratic regression model analysis was presented in Figure 1. WGR exhibited positive quadratic trends against the increased dietary lipid level, while FCR showed a negative one. The optimal dietary lipid level was estimated at 95.4 and 101.02 g/kg, based on the regression results of WGR and FCR, respectively.

### 3.2. Experiment II: Investigating the Regulatory Effects of Simvastatin in Metabolic Disorder

#### 3.2.1. Growth Performance

**Figure 2:**
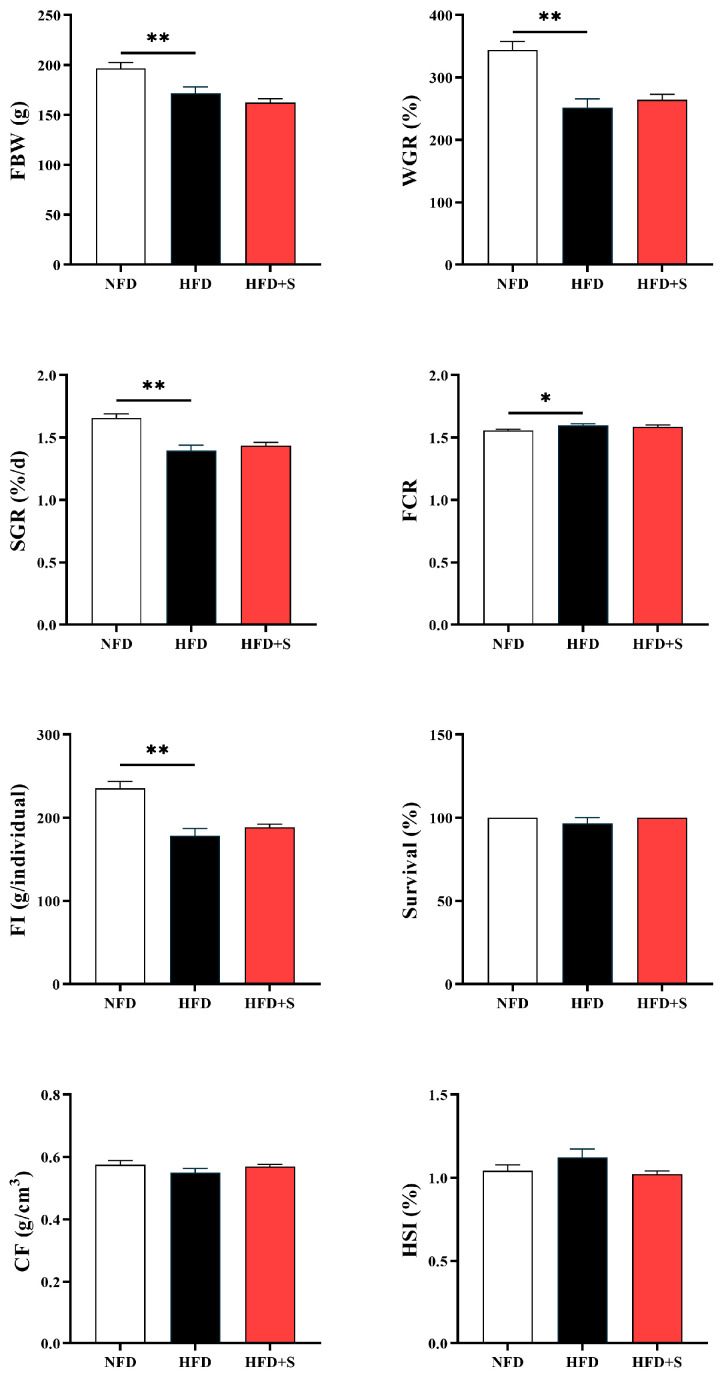
The growth performance of *A. davidianus* fed different experimental diets in experiment II. Asterisk represents significant difference (* *p* < 0.05, ** *p* < 0.01).

As shown in Figure 2, HFD feeding decreased the FBW, WGR, SGR, and FI, and increased FCR compared with the NFD group (*p* < 0.05). No significant difference could be found in survival, CF, as well as HSI (*p* > 0.05).

#### 3.2.2. Plasma Biochemical Analysis

**Figure 3:**
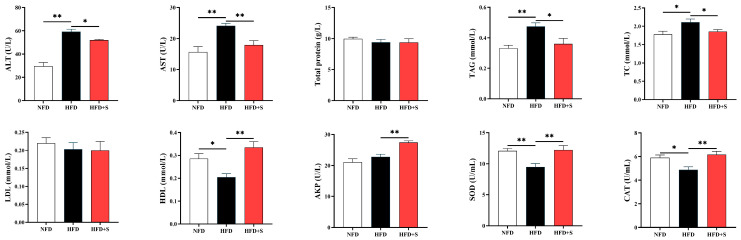
Plasma biochemical parameters of *A. davidianus* fed different experimental diets. Asterisk represents significant difference (* *p* < 0.05, ** *p* < 0.01).

Compared with the NFD group, HFD feeding increased plasma AST and ALT activities as well as TAG and TC contents, but decreased HDL content and SOD and CAT activities (*p* < 0.05, Figure 3). Compared with the HFD group, simvastatin administration reversed these phenomena and increased AKP activity at the same time (*p* < 0.05). In addition, no significant difference was noted in total protein and LDL levels (*p* > 0.05).

#### 3.2.3. Hepatic Expressions of Genes Related to Endoplasmic Reticulum Stress

**Figure 4:**
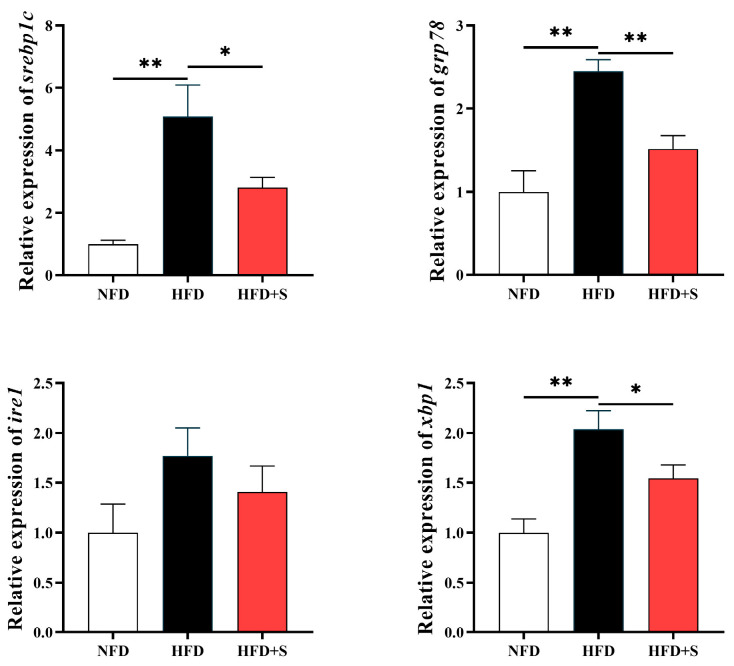
The expressions of genes related to endoplasmic reticulum stress in the liver of *A. davidianus* fed different experimental diets. Asterisk represents significant difference (* *p* < 0.05, ** *p* < 0.01).

As presented in Figure 4, the transcriptions of *srebp1c*, *grp78*, and *xbp1* in the liver were significantly enhanced by HFD (compared with the NFD group), but down-regulated by simvastatin administration (*p* < 0.05) (compared with the HFD group). However, no statistical difference was observed in the expression of *ire1* (*p* > 0.05).

#### 3.2.4. Mitochondrial Function-Related Indices

**Figure 5:**
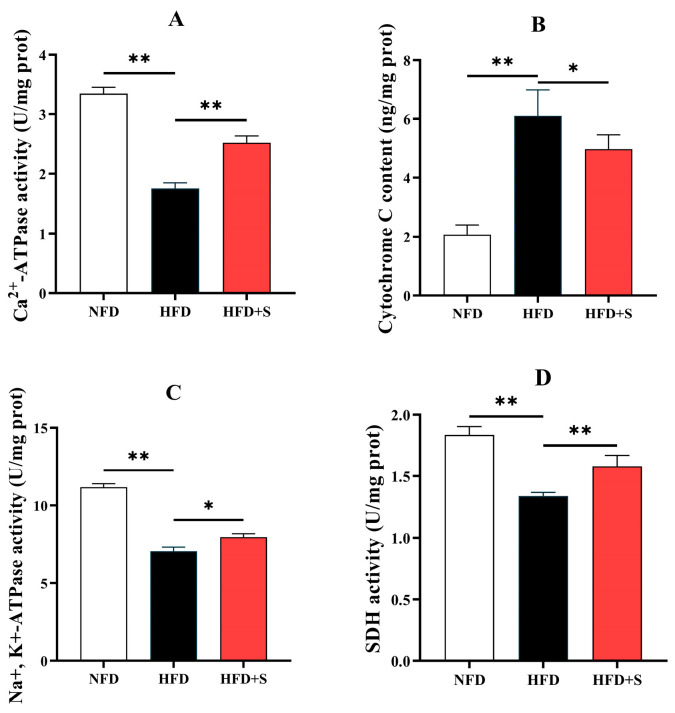
Ca^2+^-ATPase activity in the microsomes (**A**), cytochrome C content in the cytostome (**B**), Na^+^, K^+^-ATPase (**C**), and SDH (**D**) activities in the mitochondria of the liver in *A. davidianus* fed different experimental diets. Asterisk represents significant difference (* *p* < 0.05, ** *p* < 0.01).

As shown in Figure 5, compared with the NFD group, Ca^2+^-ATPase activity in the microsomes and Na^+^, K^+^-ATPase and SDH activities in the mitochondria were all markedly decreased by HFD feeding, while an opposite result was noted in cytochrome C content in cytostome. Notably, simvastatin administration remarkably reversed the above changes (*p* < 0.05) (compared with the HFD group).

## 4. Discussion

Fat is the most energy-dense nutrient with considerable impacts on the growth performance of animals [26,27]. Generally, increasing dietary fat levels within a reasonable range could promote growth performance, while excessive levels of fat could induce metabolic disorders and even growth retardation [13,28,29]. Here, the growth performance of *A. davidianus* increased significantly with increasing dietary lipid levels up to 87.9 g/kg, then decreased remarkably with further increasing lipid contents. The optimum dietary lipid level for this species is 95.16–101.02 g/kg, as was revealed by the quadratic regression analysis of WGR and FCR. This value is lower than that reported in a prior research employing the same species [27]. This might be due to the differences in feed formulation, animal management, growth stage, and genetic background of *A. davidianus* adopted in the previous and present study. Indeed, the WGR in the present study (among 211–344%) was much higher than that (among 59–106%) in the previous study [27]. However, the precise intrinsic reason for such difference needs to be investigated in the future study.

Based on the above results, the diet containing 86.8 g/kg lipid is regarded as the NFD, while a diet containing 148 g/kg lipid is designated as the HFD in the following experiment II. As expected, the HFD feeding resulted in a poor growth performance of *A. davidianus*, agreeing with the results in experiment I. The HFD-induced growth performance inhibition is proved to be highly correlated with the pathological changes in metabolic organs. As an important metabolic organ, liver largely contributes to the whole-body health and metabolism status regulation. Accordingly, the function of liver could be reflected by the plasma aminotransferase activities. In the present study, compared with the NFD group, HFD feeding elevated both ALT and AST activities in the plasma of *A. davidianus*, while plasma TAG and TC concentrations also presented similar changes. These results imply that HFD feeding damaged the liver function and disturbed lipid metabolism in this species. Notably, the administration of simvastatin effectively reduced blood lipid levels and transaminase activities compared with the HFD group, indicating its beneficial effects on lipid metabolism and liver health. These benefits might be ascribed to the inhibition of hydroxymethyl-glutaryl coenzyme A (HMG-CoA) reductase, which is responsible for catalyzing the conversion of HMG-CoA to mevalonate, and is a rate-limiting enzyme in cholesterol biosynthesis [17]. Simvastatin could inhibit hepatic HMG-CoA reductase by competing with its substrate, HMG-CoA, for the enzyme’s binding site. This action would inhibit lipid anabolism, thereby alleviating the HFD-induced lipid metabolism disorder [18]. Given this, simvastatin may improve the liver injury through regulating lipid metabolism in this study. Furthermore, an HFD feeding-induced reduction was also observed in plasma HDL level in this study, as was reversed by simvastatin supplementation. This result provides further evidence for the HFD-induced metabolic disorder and the lipid-lowering effect of simvastatin, since HDL is instrumental in transporting lipids to the liver for metabolism [30]. A similar phenomenon has also been reported in mammals, and the inner reason may be ascribed to the inhibition of HDL degradation and the enhancement of HDL synthesis [16,31].

In this study, compared with the HFD group, simvastatin administration markedly increased plasma AKP activity, implying its beneficial actions may occur beyond lipid metabolism regulation. Supportively, AKP is an important lysosomal enzyme, which plays a crucial role in metabolism, disease resistance, immune cell function, and cell damage repairing [32,33]. Therefore, the increased plasma AKP activity might indicate an enhancement of cellular immunity, anti-inflammatory functions, and tissue repair. In addition, HFD feeding decreased plasma SOD and CAT activities, as was reversed by simvastatin supplementation. This indicated that HFD can decrease the antioxidant capability of *A. davidianus*, while simvastatin can alleviate this redox imbalance. Oxidative stress is a contributor to the organ damage caused by HFD, while the antioxidant enzymes serve as the first line of defense [34]. Considering this, the liver-function-promoting effect of simvastatin could be partly attributed to the enhanced SOD and CAT activities. Additionally, as an inhibitor of HMG-CoA reductase, simvastatin inhibits the production of mevalonate, which serves as an up-stream signaling molecule of nuclear factor erythroid 2-related factor 2 (Nrf2), the major transcriptional regulator of antioxidant enzymes [35,36]. Accordingly, simvastatin might activate the transcription of the antioxidative enzymes through this pathway, thereby regulating their enzymatic activities.

As a complex membrane system, the endoplasmic reticulum (ER) is highly susceptible to oxidative stress [37]. Given its central role in protein formation, folding, modification, secretion, and lipid synthesis, oxidative damage to the ER can lead to protein unfolding and/or misfolding, thereby inducing ER stress and enhancing lipid formation [13,38]. In the current study, the transcriptions of *srebp1c*, *grp78*, and *xbp1* were all up-regulated by HFD feeding suggesting that simvastatin can inhibit the HFD-induced ER stress in *A. davidianus*. Supportively, the ER chaperone GRP78 functions as a molecular switch for the unfolded protein response. The accumulation of unfolded proteins during ER stress activates GRP78. This action would trigger the downstream signaling cascade, primarily through the splicing and activation of the transcription factor XBP1 [30,31]. However, the expression of *ire1*, which is also a key ER stress mediator [39], did not present significant difference in this study. This might be ascribed to the complex regulatory mechanism of ER stress, which contains several mediators. Accordingly, HFD might trigger ER stress in *A. davidianus* through an Ire1-independent pathway. Srebp1c has been found to be an important transcriptional regulator that contributes to lipid synthesis and acts as a down-stream factor of XBP1 [40]. There are at least two reasons to interpret these results. On the one hand, simvastatin reduces the cholesterol biosynthesis via inhibiting HMG-CoA reductase, relieving the pressure on ER metabolism [17,18]. On the other hand, simvastatin improves the antioxidative defense system, as consequently reduces the oxidative damage in ER [38].

In the present study, HFD feeding decreased Ca^2+^-ATPase activity in the microsomes, suggesting an impaired ability of ER to regulate Ca^2+^ homeostasis. Supportively, microsomes are fragments of ER [41], and ER Ca^2+^-ATPase is responsible for the uptake of Ca^2+^ from cytostome [42]. According to a previous study, this phenomenon has been reported to be a consequence of the ER stress [43]. Moreover, it has been reported that ER stress exerts cytotoxic effects by inducing apoptosis. Mechanistically, the deregulated Ca^2+^ homeostasis caused by ER stress would induce mitochondrial Ca^2+^ overload. This would damage the metabolic function of mitochondria, leading to the promoted release of the respiratory chain component-cytochrome C to cytoplasm, thereby triggering the apoptosis death cascade [44]. Accordingly, HFD reduced Na^+^, K^+^-ATPase, and SDH activities in the mitochondria and increased cytostome cytochrome C concentration in this study. These results support that HFD feeding causes hepatocyte damage via ER stress. Na^+^, K^+^-ATPase is critical in maintaining ion homeostasis, while SDH plays dual roles in tricarboxylic acid cycle and oxidative phosphorylation. The reduced mitochondrial Na^+^, K^+^-ATPase, and SDH activities indicated mitochondrial dysfunction [45,46]. Furthermore, simvastatin administration mitigated all the aforementioned adverse phenomena. This may be attributed to the inhibited ER stress, which improves ER function and cellular Ca^2+^ homeostasis, thereby enhancing mitochondrial function.

## 5. Conclusions

Collectively, the present study showed that the optimal lipid level in the diet of *A. davidianus* is 95.16–101.02 g/kg. Dietary lipid levels up to 148 g/kg would reduce growth performance and result in metabolic disorders in this species. Adding 0.1 g/kg simvastatin could positively regulate the growth and metabolic status of *A. davidianus* fed the HFD, as may be achieved by ER stress inhibition and mitochondrial function enhancement.

## Figures and Tables

**Table 1:** Formulation and proximate composition of the experimental diets in experiment I (g kg^−1^, dry matter).

Ingredients ^1^	Dietary Lipid Levels (g/kg)				
	32.8	58.7	87.9	122.4	149.2
Fish meal	210	210	210	210	210
Soybean meal	200	200	200	200	200
Casein	166.3	166.3	166.3	166.3	166.3
Gelatin	50	50	50	50	50
Wheat middling	180	149.1	118.3	87.4	56.6
Gelatinized starch	150	150	150	150	150
Soybean oil	1.35	16.8	32.2	47.65	63.05
Fish oil	1.35	16.8	32.2	47.65	63.05
Choline chloride	3	3	3	3	3
Calcium biphosphate	18	18	18	18	18
Premix ^2^	20	20	20	20	20
Proximate composition					
Moisture	95.2	97.4	96.3	94.7	95.1
Crude protein	448.7	453.5	452.2	449.4	451.4
Crude lipid	32.8	58.7	87.9	122.4	149.2
Ash	97.4	94.2	98.7	100.2	95.8

^1^ All ingredients were purchased from Haipurui Feed Co., Ltd., Nanjing, China. ^2^ Premix provided the following nutrients in per kilogram of the experimental diet: CuSO_4_·5H_2_O, 2.0 g; FeSO_4_·7H_2_O, 25 g; ZnSO_4_·7H_2_O, 22 g; MnSO_4_·4H_2_O, 7 g; Na_2_SeO_3_, 0.04 g; KI, 0.026 g; CoCl_2_·6H_2_O, 0.1 g; Vitamin A, 900,000 IU; Vitamin D, 200,000 IU; Vitamin E, 4500 mg; Vitamin K_3_, 220 mg; Vitamin B_1_, 320 mg; Vitamin B_2_, 1090 mg; Vitamin B_5_, 2000 mg; Vitamin B_6_, 500 mg; Vitamin B_12_, 1.6 mg; Vitamin C, 5000 mg; Pantothenate, 1000 mg; Folic acid, 165 mg; Choline 60,000 mg, the same as blow.

**Table 2:** Formulation and proximate composition of the experimental diets in experiment II (g kg^−1^, dry matter).

Ingredients	Groups		
	NFD	HFD	HFD_S
Fish meal	210	210	210
Soybean meal	200	200	200
Casein	166.3	166.3	166.3
Gelatin	50	50	50
Wheat middling	118.3	56.6	56.5
Gelatinized starch	150	150	150
Soybean oil	32.2	63.05	63.05
Fish oil	32.2	63.05	63.05
Choline chloride	3	3	3
Calcium biphosphate	18	18	18
Premix	20	20	20
Simvastatin	0	0	0.1
Proximate composition			
Moisture	95.7	94.8	97.7
Crude protein	451.6	453.2	452.1
Crude lipid	86.8	148.4	147.3
Ash	96.8	97.7	96.5

**Table 3:** The sequences of the primers used in the present study.

Gene Abbreviations	Gene Full Names	Primer Sequences (5′–3′)	Source	Amplicon Sizes
*srebp1c*	sterol regulatory element-binding protein-1c	ACTCGGTGTGGATATCGT	PRJNA1332065	105 bp
		TGAACGCAATCTGGAAG		
*grp78*	glucose-regulated protein 78	TTACAGACCTTAGAGACTG	PRJNA1332065	122 bp
		GGGGGTTGATCCACTCTAT		
*ire1*	inositol-requiring enzyme 1	ATCGAATCAGACGCTGACA	PRJNA1178345	155 bp
		CCATCTGCACTCTAGCCATC		
*xbp1*	x-box binding protein 1	ACACGGCACTAGGGGCACTC	PRJNA1178345	107 bp
		AGCTCATCGCTTGGATCTGGG		
*β-actin*	-	CTTGTATTCTCAGCAAGAC	HQ822274.1	135 bp
		CATCTAGCCTCCATTACC		

**Table 4:** The growth performance of *A. davidianus* fed diets containing different lipid levels in experiment I.

Parameters	Dietary Lipid Levels (g/kg)				
	32.8	58.7	87.9	122.4	149.2
FBW ^1^	137.56 ± 5.94 ^a^	171.37 ± 4.31 ^bc^	196.44 ± 6.25 ^c^	171.75 ± 6.3 ^bc^	155.28 ± 5.66 ^ab^
WGR ^2^	211.87 ± 13.40 ^a^	286.99 ± 11.40 ^bc^	344.15 ± 13.70 ^c^	289.21 ± 15.48 ^bc^	251.77 ± 14.20 ^ab^
SGR ^3^	1.26 ± 0.05 ^a^	1.50 ± 0.03 ^bc^	1.66 ± 0.03 ^c^	1.51 ± 0.04 ^bc^	1.40 ± 0.04 ^ab^
FCR ^4^	1.64 ± 0.02 ^b^	1.60 ± 0.01 ^ab^	1.56 ± 0.01 ^a^	1.58 ± 0.01 ^a^	1.60 ± 0.01 ^ab^
FI ^5^	153.26 ± 11.26 ^a^	202.69 ± 5.97 ^bc^	237.24 ± 9.24 ^c^	200.87 ± 10.61 ^bc^	177.56 ± 8.08 ^ab^
Survival ^6^	96.67 ± 3.33	93.33 ± 3.33	96.67 ± 3.33	90.00 ± 5.77	96.67 ± 3.33
CF ^7^	0.56 ± 0.01	0.56 ± 0.01	0.57 ± 0.01	0.55 ± 0.01	0.55 ± 0.02
HSI ^8^	4.74 ± 0.15 ^a^	4.74 ± 0.12 ^a^	5.07 ± 0.12 ^ab^	5.30 ± 0.08 ^bc^	5.59 ± 0.09 ^c^

Values in the same row with the same superscripts mean no significant difference (*p* > 0.05). ^1^ Final body weight (FBW, g). ^2^ Weight gain rate (WGR, %) = (final body weight − initial body weight)/initial body weight × 100. ^3^ Specific growth rate (SGR, %/d) = (ln final body weight − ln initial body weight)/feeding days × 100. ^4^ Feed conversion ratio (FCR) = dry feed intake/wet weight gain. ^5^ Feed intake (FI, g/individual). ^6^ Survival (%) = number of live individuals at the end of feeding trial/initial individuals’ number × 100. ^7^ Condition factor (CF, g/cm^3^) = individual body weight/body length^3^. ^8^ Hepatosmatic index (HSI) = individual liver weight/body weight.

## Data Availability

The data presented in this study are available on request from the corresponding author.
